# Subacute Combined Degeneration in a Young Female Patient After Sleeve Gastrectomy

**DOI:** 10.7759/cureus.50336

**Published:** 2023-12-11

**Authors:** Ali Al Ani, Pir Abdul Ahad Aziz Qureshi, Vikram Rao Bollineni

**Affiliations:** 1 Department of Radiology, UZ Brussels University Hospital, Brussel, BEL; 2 Department of Radiology, Landspítali - The National University Hospital of Iceland, Reykjavík, ISL; 3 Department of Radiology, UZ Brussels University Hospital, Brussels, BEL

**Keywords:** subacute combined degeneration, subacute combined degeneration of the spinal cord, vitamin b12 deficiency symptoms, vit b12 deficiency, spinal cord

## Abstract

The neurological symptoms of vitamin B12 deficiency are variable but primarily consist of combined spinal sclerosis, peripheral neuropathy, and dementia. Movement disorders and cerebellar ataxia are reported less frequently. We present a case of a young female patient with vitamin B12 deficiency after sleeve gastrectomy, resulting in subacute combined degeneration of the spinal cord (SACD).

## Introduction

B12 is an essential nutrient for proper neurological function. It is critical for the synthesis of myelin, the preservation of neuronal integrity, and the regulation of neurotransmitters [1]. Inadequate vitamin B12 intake is an uncommon cause of vitamin B12 deficiency; however, it is the most common cause among devout vegetarians. Pernicious anemia, malabsorption syndrome (e.g., bacterial proliferation of the small intestine), regional enteritis, or surgical procedures (e.g., gastric operations or ileal resection) are the most frequent causes of B12 deficiency [2]. The rare and diverse initial neurologic manifestations of B12 deficiency arrays complicate the diagnostic process. It is critical to initiate the replacement therapy as soon as the symptoms appear to prevent the development of neurological complications. We present a case of vitamin B12 deficiency in a 26-year-old female patient who was diagnosed with subacute combined degeneration of the spinal cord (SACD) after sleeve gastrectomy. The laboratory and magnetic resonance imaging (MRI) results corroborated in making this diagnosis.

## Case presentation

A 26-year-old woman was referred to the emergency department for a one-week history of gait disorders and sensation disturbances in the lower limbs, which extended later to the upper limb. She has a feeling that all her extremities are very cold. In previous history, she had a sleeve gastrectomy about one year ago. Subsequently, she was advised to take multivitamin supplements but was non-compliant.

The general physical examination showed a pale patient with a blood pressure of 118/60 mm Hg, a heart rate of 116 beats/minute, and a temperature of 36.9°C. Neurological examination revealed a jerky gait having positive Romberg's sign. There was a decreased sensation in all four limbs, mainly on the internal side, impaired two-point discrimination, and vibration sense. The deep tendon reflexes, including patellar, Achilles, biceps, and triceps tendon reflexes, showed hyperreflexia. The Babinski reflex was also positive bilaterally. The complete blood count (CBC), vitamin B12 and folic acid, and homocysteine levels were performed, which revealed macrocytic anemia with decreased vitamin B12 and folic acid levels and increased homocysteine levels.

**Figure 1:**
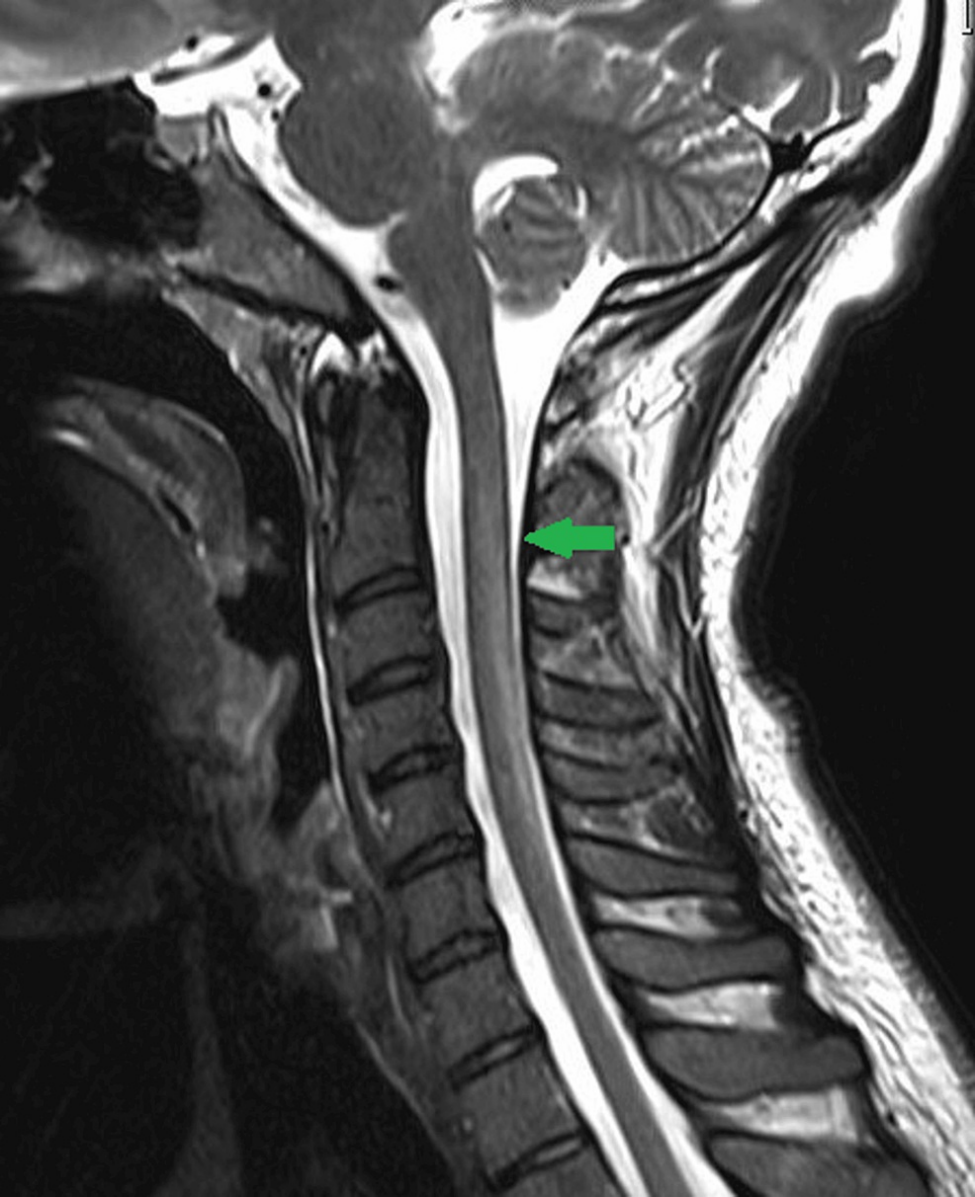
MRI cervical spine T2 weighted image (sagittal view) T2 hyperintense signals can be seen within the posterior part of the cervical cord, extending from C2 to C6 vertebral levels and associated with mild swelling of the cervical cord (green arrow). MRI, magnetic resonance imaging

Computed tomography (CT) of the brain was normal. Subsequently, an MRI of the spinal cord was performed for further evaluation of the patient's symptoms, which showed T2 hyperintense signals involving the posterior part of the spinal cord extending from the level of C2 to C6 and associated with mild swelling of the cervical cord on sagittal view (Figure 1).

**Figure 2:**
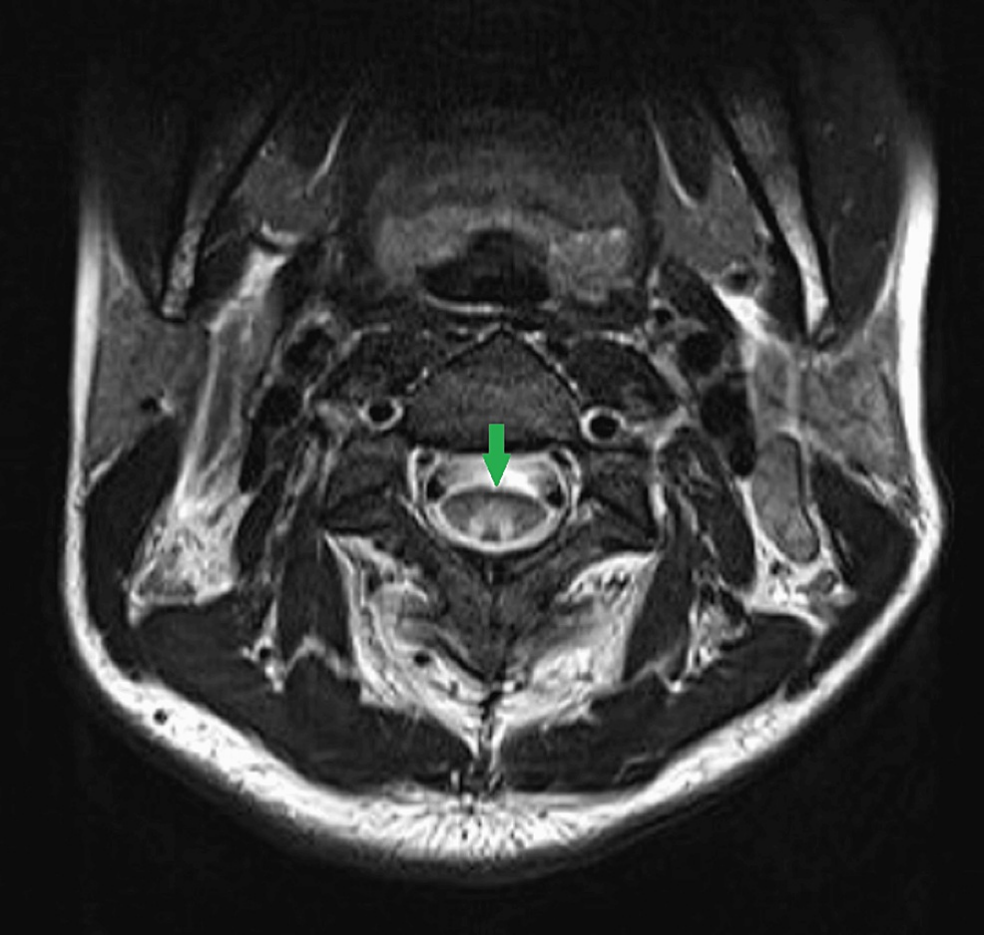
MRI cervical spine T2 weighted image (axial view) Symmetric T2 hyperintense signals within the posterior spinal cord give an inverted V appearance (green arrow).

On axial T2 images, there were symmetric T2 hyperintense signals within the posterior spinal cord, corresponding to the dorsal columns giving a characteristic "inverted V" or "inverted rabbit ears" appearance, which is a characteristic sign of SACD (Figure 2).

## Discussion

A variety of neurologic complications may result from a vitamin B12 deficiency, including SACD of the spinal cord. The production of gastric acid and intrinsic factors in the stomach plays a crucial role in the absorption of vitamin B12. By eliminating epithelial cells during various bariatric procedures, including sleeve gastrectomy and Roux-en-Y gastric bypass, the production of these agents can be substantially diminished. Our patient failed to adhere to a consistent vitamin supplement regimen, which is essential for maintaining an average level of vitamin B12. Vitamin B12 deficiency results in the failure of the conversion of methyl malonyl coenzyme A to succinyl coenzyme, resulting in a significant accumulation of methylmalonic acid in the myelin material of the spinal cord. The dorsal columns, located in the posterior funiculus of the spinal cord, are the preferred target of this process [3]. Consequently, SACD is also referred to as "funicular myelosis."

Typical initial symptoms include paresthesia of the extremities (hands and feet). Over time, the condition may advance to gait ataxia, distal weakness, and sensory loss, especially in the lower extremities. If untreated, the disease may result in the development of ataxic paraplegia [4].

Radiologically, on MRI, SACD usually presents as T2 hyperintense signals within the dorsal column of the spinal cord extending to multiple vertebral levels and moderate swelling of the cervical and thoracic cord, as seen in our case. On axial images, the T2 hyperintense signals can be seen involving the posterior funiculus of the spinal cord symmetrically; thereby, giving an inverted V appearance [5]. Although this sign is characteristic of SACD, MRI findings should be correlated with the medical history and clinical examination to exclude other common diseases of the spinal cord like multiple sclerosis, infectious and inflammatory diseases (e.g., HIV vascular myelopathy, herpes viruses, and sarcoidosis), ischemia, and neoplasms [6]. Injectable vitamin B12 supplementation should be arranged without delay in such cases as failure to do so may result in irreversible neurologic injury.

## Conclusions

Patients with sleeve gastrectomy and Roux-en-Y gastric bypass surgeries have a higher predilection for B12 deficiency in cases of improper intake of vitamin supplements. The inverted V sign is a typical characteristic feature of SACD on MRI, and the reporting radiologists must be aware of this sign to make a quick and appropriate diagnosis followed by immediate treatment to avoid irreversible disability.
